# Web-Based Discussion and Illicit Street Sales of Tapentadol and Oxycodone in Australia: Epidemiological Surveillance Study

**DOI:** 10.2196/29187

**Published:** 2021-12-20

**Authors:** Joshua Black, Zachary R Margolin, Gabrielle Bau, Richard Olson, Janetta L Iwanicki, Richard C Dart

**Affiliations:** 1 Rocky Mountain Poison and Drug Safety Denver, CO United States; 2 Department of Emergency Medicine University of Colorado Hospital Aurora, CO United States

**Keywords:** Australia, opioids, web-based discussion, diversion

## Abstract

**Background:**

Opioid use disorder and its consequences are a persistent public health concern for Australians. Web activity has been used to understand the perception of drug safety and diversion of drugs in contexts outside of Australia. The anonymity of the internet offers several advantages for surveilling and inquiring about specific covert behaviors, such as diversion or discussion of sensitive subjects where traditional surveillance approaches might be limited.

**Objective:**

This study aims to characterize the content of web posts and compare reports of illicit sales of tapentadol and oxycodone from sources originating in Australia. First, post content is evaluated to determine whether internet discussion encourages or discourages proper therapeutic use of the drugs. Second, we hypothesize that tapentadol would have lower street price and fewer illicit sales than oxycodone.

**Methods:**

Web posts originating in Australia between 2017 and 2019 were collected using the Researched Abuse, Diversion, and Addiction-Related Surveillance System Web Monitoring Program. Using a manual coding process, unstructured post content from social media, blogs, and forums was categorized into topics of discussion related to the harms and behaviors that could lead to harm. Illicit sales data in a structured format were collected through a crowdsourcing website between 2016 and 2019 using the Researched Abuse, Diversion, and Addiction-Related Surveillance System StreetRx Program. In total, 2 multivariable regression models assessed the differences in illicit price and number of sales.

**Results:**

A total of 4.7% (28/600) of tapentadol posts discussed an adverse event, whereas 10.27% (95% CI 9.32-11.21) of oxycodone posts discussed this topic. A total of 10% (60/600) of tapentadol posts discussed unsafe use or side effects, whereas 20.17% (95% CI 18.92-21.41) of oxycodone posts discussed unsafe use or side effects. There were 31 illicit sales reports for tapentadol (geometric mean price per milligram: Aus $0.12 [US $0.09]) and 756 illicit sales reports for oxycodone (Aus $1.28 [US $0.91]). Models detected no differences in the street price or number of sales between the drugs when covariates were included, although the potency of the pill significantly predicted the street price (*P*<.001) and availability predicted the number of sales (*P*=.03).

**Conclusions:**

Australians searching the web for opinions could judge tapentadol as safer than oxycodone because of the web post content. The illicit sales market for tapentadol was smaller than that of oxycodone, and drug potency and licit availability are likely important factors influencing the illicit market.

## Introduction

### Background

With a steady increase in opioid prescribing [1], opioid use disorder and its consequences are a persistent public health concern for Australians despite the lack of national research on the topic. The few papers presenting national data show that most people who inject drugs use opioids [2], and opioid deaths rose through 2012 [3]. Prescription opioids continue to be misused, and the prevalence of use differs for each drug [2]. The most recent reports show that ambulance attendances involving prescription opioids are rising in Victoria, Australia [4]. Diversion, which is the sale or acquisition of a controlled substance outside the regulatory system, contributes to serious medical consequences. In other parts of the world, there is evidence that at least one-fourth of opioid overdose deaths involve a diverted drug [5]. Without the direction of a health care provider to provide safe use information and guidance (such as using multiple drugs at once), individuals using diverted drugs could be at higher risk of adverse events, such as fatality. Understanding the availability of diverted drugs and desirability differences between these drugs will help guide effective policies to control drug harms and create postmarketing systems that monitor safety and efficacy across all populations of users. Given the relatively limited country-wide surveillance, evaluation of data from internet sources could provide an initial understanding of how Australians perceive the safety of drugs and how frequently a drug is sold outside of the licit drug distribution channels. Investigations of street sale volume and web-based discussion of drug misuse together provide complementary perspectives on the desirability of drugs in the Australian market.

The anonymity of the internet offers several advantages for surveilling and inquiring about specific covert behaviors, such as diversion or discussion of sensitive subjects, where traditional surveillance approaches might be limited. Crowdsourcing of street prices has been used to discern factors that influence differences in prices between oxycodone and oxymorphone [6], identify unmet needs for buprenorphine assisted opioid therapy [7], and demonstrate the rarity of tapentadol diversion relative to other controlled drugs [8]. The monitoring of blogs and forums and social media has shown divergent trends in the discussion of addiction and overdose between drugs [9], tracked the popularity of marijuana concentrates [10], and identified tampering methods for reformulated oxycodone [11].

### Objectives

The aim of this study is to characterize safety-related web post content and compare reports of illicit sales of tapentadol and oxycodone products from sources originating in Australia. Tapentadol is a relatively new drug in Australia and was first approved in 2011. It is possible that the diversion and desirability of a new drug are different from those of drugs with a longer history and larger market availability, such as oxycodone. First, these 2 drugs were compared to describe the differences in web-based discussions of serious health consequences. The textual content of the posts was evaluated to determine whether internet discussion encouraged or discouraged the proper therapeutic use of the drugs. Second, 2 hypotheses were tested using street sales data. As patients exposed to tapentadol have lower odds of physician shopping [12], we hypothesized that tapentadol would have lower street prices and fewer illicit sales. Differences between drugs were modeled with covariates to account for other effects on sales.

## Methods

### Overview

The Researched Abuse, Diversion, and Addiction-Related Surveillance System is a compilation of individual data collection programs that monitor drug use–related outcomes and behaviors. In total, 2 systems, the Web Monitoring Program and the StreetRx Program, were used to characterize web posts and reports of street sales involving tapentadol or oxycodone in Australia. The Web Monitoring Program, established in 2014, focuses on the collection and organization of real-time web content about prescription drugs from >150 million websites on the internet, including social media, blogs, and forums. The StreetRx Program collects crowdsourced information related to illicit street sales of drugs. StreetRx users select drugs purchased or sold from a drop-down menu of substances and formulations (pill or tablet, syrup or liquid, film, patch, etc). All statistical analyses were conducted using SAS (version 9.4; The SAS Institute).

### Data Collection and Analysis of Internet Posts

Methods of data collection, cleaning, and estimation procedures for the Web Monitoring Program have been described elsewhere [9]. Briefly, all data were collected using a web-crawling platform (Salesforce.com Inc) that scrapes data from public websites that permit content viewing by a third party. Examples of sites that permit this type of crawling include Twitter, Reddit, public blogs and forums, and comment sections on many news sites. Private sites, such as personal Facebook pages, Bluelight, and other password-protected sites do not permit this type of crawling. For this study, posts mentioning either tapentadol or oxycodone were collected from websites that permit public scraping of data, and the weekly number of posts was calculated. Posts mentioning tapentadol or oxycodone were identified based on specified search-string criteria (such as drug name, associated misspellings, product names, and unique slang terms). The keywords for each drug substance and product were generated using a phonetic algorithm and then validated using the number of hits when entered into a common search engine. Other keywords were identified during the manual coding process. The search strings used are listed in Multimedia Appendix 1. Posts between 2017 and 2019 were collected, and only posts that originated in Australia as determined by the IP address of the post were included in this study. Nonsubstantive posts, such as nonsensical posts or posts from web-based pharmacies, were removed. The remaining posts had substantive content about a person’s experience, opinion, or understanding of the drugs. The content of the posts was manually categorized into topics by a team of trained coders, who pass at least a 90% biannual interrater reliability test [9], into a series of safety-related topics based on the definitions listed in Textbox 1. Grounding theory was previously used to develop topics and definitions [11]. In brief, topics were identified by trained researchers reviewing samples of the posts for emergent topics. These data-driven topics were identified in a 2-round process, and a standard definition was created for coding purposes. The percentages of posts discussing each topic and 95% CIs were calculated. All scraped posts mentioning tapentadol were coded. Therefore, the statistics presented here are exact and represent the total volume of tapentadol posts from Australia during the period. Owing to the volume of scraped posts mentioning oxycodone, a sample of posts was coded. A stratified, random sample without replacement and with proportional allocation was obtained from the population of identified posts. Strata included both time (by week) and origin (social media or blogs and forums) of the web posts. Therefore, statistics for oxycodone are estimates presented with a 95% CI.

**Coding definitions for web posts.**

**Coding definitions**

- Abuse
  A mention that indicates the use of a drug to gain a high, euphoric effect or some other psychotropic effect
- Addiction—a mention that indicates one or more of the following:
  Psychological or physical dependence on a drug
  Tolerance to the psychotropic effects of a drug
  Withdrawal effects when discontinuing use of a drug
- Adverse event
  Any untoward medical occurrence in a patient or clinical investigation participant (individual) administered a medicinal product and which does not necessarily require to have a causal relationship with this treatment. An adverse event can therefore be any unfavorable and unintended sign (eg, an abnormal laboratory finding), symptom, or disease temporally associated with the use of a medicinal product, whether considered related to this medicinal product or not.
  Adverse events include misuse, abuse, overdose, death, drug dependency, side effect, exposure during pregnancy, exposure during breastfeeding, medication error, lack of effect, off-label use, suspected transmission of infectious agents, quality defect or falsified medicine (counterfeit product), and occupational exposure.
- Concomitant use
  The concurrent administration of 2 or more substances of interest such that the effects of the substances overlap
- Death
  A mention that indicates that death has occurred because of a drug of interest
- Injection or intranasal administration
  A mention that discusses the route of administration for a drug, defined as the path by which a drug is taken into the body
- Overdose
  A mention that indicates the accidental or intentional overdose of a drug using a dangerous amount of a drug (ie, a quantity greater than that recommended or generally prescribed) or use which may result in a medical intervention
- Post
  A single point of communication entered by 1 individual at 1 specific time point
- Tampering with product
  A mention of a drug that discusses manipulating a product formulation to change its drug delivery in a way not specified by the manufacturer

All posts were categorized as conveying negative, positive, or neutral sentiment. Sentiment was defined as the dominant view or opinion of a drug within the post. Positive sentiments promoted the therapeutic benefits or safe use of a drug. Negative sentiments encouraged unsafe or inappropriate use of a drug or reported ineffectiveness or side effects. Positive and negative sentiments were further broken down into mutually exclusive sentiment categories. Positive sentiment must have (1) promoted therapeutic benefit, (2) discouraged abuse, or (3) referred to a product in another positive way. Negative sentiment must have (1) discouraged therapeutic benefit, (2) promoted abuse, or (3) referred to a product in another negative way (eg, side effects). Posts categorized as having neutral sentiment were those in which no predominant opinion existed, or the sentiment could not be determined. Posts were categorized as part of ongoing data collection using the Researched Abuse, Diversion, and Addiction-Related Surveillance System; therefore, coders were blind to the study purpose. The content of the posts was redacted to preserve the anonymity of the authors. The content of each post was changed so that the post could not be found using web-based search engines, but it did not alter the message or context of the post. Changes included the correction of grammar and spelling, replacement of words with synonyms, and reordering of sentence structure.

### Street Sales Data Collection and Analysis

Methods of data collection for the StreetRx Program have been described elsewhere [13]. In brief, a website with an Australian domain name collected product identity, geographic location, and sale price in Australian dollars, as entered by the website user. Reports of illicit street sales were entered by website users who had either participated in the transaction or heard about the prices. Website users were prompted to select the drug name from a list of licit and illicit drugs sold in Australia and were required to enter the Australian state or territory where the sale occurred. The website also requires the user to enter the price paid per unit, dose, and date of the transaction. The price per milligram is calculated from the price paid per unit and the dose. Website users are not compensated for entering information; however, they are shown a list of other sales for the same drug that occurred in the same area before entering the data. Sales data for tapentadol and oxycodone were collected from 2016 to 2019. For summarizing in this report, prices were converted to US dollars at a conversion of Aus $1 to US $0.71, which was the conversion on December 1, 2021.

The number of reports, geometric mean of the sale price per milligram, and percentages of release type and reason for sale were calculated. The geometric mean was used as the distribution of prices reported was not symmetrical, and it better represented the central tendency than the arithmetic mean [6].

Regression models were run to test 2 separate hypotheses. First, we hypothesized that there was a difference in price between oxycodone and tapentadol. Differences in price were tested using a linear multivariable model while controlling for the year the sale occurred. The potency of the drug in morphine milligram equivalents (MME) was added separately to the simplified model to test if differences in price were predicted by potency. The outcome for the first hypothesis was a log-transformed price. Conversion factors for MME from the United States Centers for Disease Control and Prevention were used [14]. Influential points were defined as a Cook distance >0.01. Second, we hypothesize that there is a difference in the number of sales between oxycodone and tapentadol. The difference in the likelihood of reporting a sale was tested using a Poisson multivariable model while controlling for general site use. Standard units sold (ie, licit availability) were added separately to test if differences in the number of illicit sales were predicted by licit availability. The outcome in the second model was the number of quarterly reports. Standard units sold are estimates of the number of drug units sold from manufacturers to dispensing outlets (eg, pharmacies and supermarkets); data were obtained from IQVIA. Estimates of standard units sold were used to control for the amount of drug available for diversion. Drug availability data were only obtained from 2016 to 2018; therefore, the models for the second hypothesis did not include reports from 2019.

## Results

### Characteristics of Web-Based Discussion

**Figure 1:**
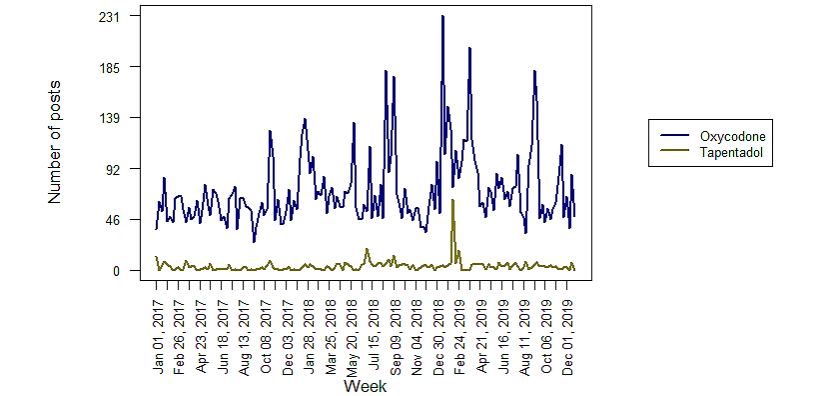
Weekly number of web posts from Australian sources: total number of posts per week that mention tapentadol and oxycodone.

The weekly number of posts for tapentadol and oxycodone were steady across the study period, with a potential increase in posts in early 2019 (Figure 1). A total of 17,634 oxycodone posts were collected for sampling and 695 tapentadol posts were collected for coding. After coding to remove nonsubstantive posts, there were 600 substantive tapentadol posts originating in Australia. After sampling and coding, an estimated 8598 (95% CI 8456-8739) substantive oxycodone posts were identified. The percentages of posts on discussion topics are listed in Table 1. Most of the posts shared an experience or opinion regarding the drug. An adverse event was discussed in 4.7% (28/600) of tapentadol posts and 10.27% (95% CI 9.32-11.21) of oxycodone posts. A total of 2.7% (16/600) of tapentadol posts and 5.42% (95% CI 4.72-6.12) of oxycodone posts discussed addiction. Discussion of concomitant use was higher among tapentadol posts than among oxycodone posts. The use of another drug was discussed in 14.7% (88/600) of tapentadol posts compared with 6.05% (95% CI 5.31-6.79) of oxycodone posts. When tapentadol was concomitantly used, paracetamol, oxycodone, and pregabalin were the 3 most frequently used second drug. When oxycodone was concomitantly used, paracetamol, morphine, and tramadol were the 3 most frequently used second drug. For either drug, <1% of posts discussed abuse, overdose, death, product tampering, injection of the product, or intranasal administration of the product.

**Table 1:** Web-based discussion topics (2017-2019).

Characteristics		Tapentadol^a^	Oxycodone
Number of substantive posts, n (95% CI)		600	8598 (8456-8739)
**Discussion topic, % (95% CI)**			
	Sharing experience or opinion	98.33	99.79 (99.64-99.94)
	Seeking information	3.33	1.16 (0.83-1.50)
	Abuse	0.17	0.79 (0.52-1.07)
	Overdose	0.17	0.38 (0.19-0.57)
	Addiction	2.67	5.42 (4.72-6.12)
	Death	0.00	0.79 (0.51-1.07)
	Adverse events	4.67	10.27 (9.32-11.21)
	Concomitant use with another product	14.70	6.05 (5.31-6.79)
	Tampering with product	0.00	0.05 (0.00-0.13)
	Injection of product	0.17	0.06 (0.00-0.14)
	Intranasal administration of product	0.00	0.19 (0.05-0.33)
**Overall sentiment, % (95% CI)**			
	Positive	16.83	9.87 (8.94-10.80)
	Negative	10.00	20.17 (18.92-21.41)
	Neutral	73.17	69.96 (68.54-71.39)
**Positive sentiment,^b^ % (95% CI)**			
	Promoting therapeutic use	50.50	27.70 (23.25-32.15)
	Discouraging abuse	0.00	0.96 (0.02-1.89)
	Others	50.50	71.81 (67.34-76.28)
**Negative sentiment,^b^ % (95% CI)**			
	Discouraging therapeutic use	0.00	0.00 (0.00)
	Promoting abuse	6.67	20.91 (18.10-23.72)
	Others	95.00	82.12 (79.48-84.77)

^a^All tapentadol posts were coded, and therefore, values are exact; CIs are not applicable.

^b^Posts could be identified with more than one discussion topic or sentiment; therefore, percentages would not sum to 100% within each category.

The overall sentiment of tapentadol posts was more positive and less negative than that of oxycodone posts (Table 1). Among positive sentiment posts, a higher percentage of posts encouraged the therapeutic benefit of tapentadol than that of oxycodone. A total of 50.5% (51/101) of positive tapentadol posts encouraged therapeutic benefit compared with 27.7% (95% CI 23.25-32.15) of positive oxycodone posts. Among negative posts, a lower percentage of posts promoted abuse of tapentadol compared with that of oxycodone. A total of 7% (4/60) of negative tapentadol posts encouraged abuse compared with 20.91% (95% CI 18.10-23.72) for negative oxycodone posts. Side effects were more prevalent among negative tapentadol posts. A total of 40% (24/60) of negative tapentadol posts and 24.21% (95% CI 21.23-27.18) of negative oxycodone posts discussed side effects.

Representative redacted posts demonstrate coding practices for topics and sentiments (Table 2). Both simple and challenging posts are shown here. Nearly all posts involved the author of the post sharing an experience or opinion of the drugs. Some posts were clear in how the author of the post intended to use the drug. Posts 1, 2, and 3 show how the coding of topics was conducted. Post 1 demonstrates a post coded for sharing an experience but not coded for abuse. In post 1, the intention was clearly suicidal and not to gain a high, euphoric effect or some other psychotropic effect. In post 2, a clear description of drug-related death of a person was provided. In post 3, the author of the post indicates the intention to use in the future to “not feel.” Given that the activity is in the future, it was challenging to assign intention to the post. Ultimately, this post was not assigned to abuse, given the ambiguity. Posts 4, 5, and 6 show differences in how sentiment was assigned; challenging posts are shown here to demonstrate how sentiment might be assigned and to demonstrate the pitfalls in the manual coding of drug-related sentiment. In post 4, the author of the post discussed the use of a tapentadol product without concomitant use, switched to another opioid product, and encouraged others to use tapentadol. This post was coded as promoting the safe use of the drug (ie, positive sentiment). In post 5, the author of the post discussed sickness from combining drugs. This post was coded as describing a negative effect from use (ie, negative sentiment). In post 6, the author of the post took an excessive amount of the drug, thinking it was a different drug, and the author of the post indicated that the experience was *pretty interesting.* This post was coded as promoting unsafe use of the drug (ie, negative sentiment).

**Table 2:** Redacted, representative posts from web-based discussion.

Redacted post			Coded topics		Coded sentiment
**Oxycodone**					
	Post 1: “I’m 30 now, and when I was 28 I tried my hardest to end my life. I took a pack of oxycontin on a ten story balcony. The next day, I woke up covered in vomit under a piano. I didn’t tell a soul because it seemed dumb.”	Sharing experience		Negative	
	Post 2: “I’ve just heard that the husband of my cousin, who is only in his 30s, had pneumonia and died in his sleep. A doctor gave him oxycodone for muscle pain in his chest. The oxycodone suppressed his breathing and he just stopped breathing in his sleep.”	Sharing experience and death		Negative	
	Post 3: “I am emotional, plus my dog just puked on the rug. I’m going to drink wine and take oxycodone until I can’t feel.”	Sharing experience		Negative	
**Tapentadol**					
	Post 4: “I’ve been off tapentadol for four months now and the withdrawal was terrible, but I put up with the pain and went down to Tramadol SR. The pain relief is sometimes not worth the mental price. I hope tapentadol works out for you.”	Sharing experience and addiction		Positive	
	Post 5: “Throughout the day I had a few glasses of white wine which caused me to be sick at night. I’ve realized that Alcohol with Clonazepam or Tapentadol does not mix well together. Clonazepam is only potent at a low dose and about 0.5 mg of it equals about 10 mg of Diazepam.”	Sharing experience and concomitant use (alcohol and clonazepam)		Negative	
	Post 6: “Today has been pretty interesting! Here’s a suggestion: store medicines that look similar in different locations not near each other. I took tapentadol thinking it was nizatidine. I had 500 mg of tapentadol in my body, all at the same time, and that is a lot!”	Sharing experience		Negative	

### Characteristics of Street Sales

**Figure 2:**
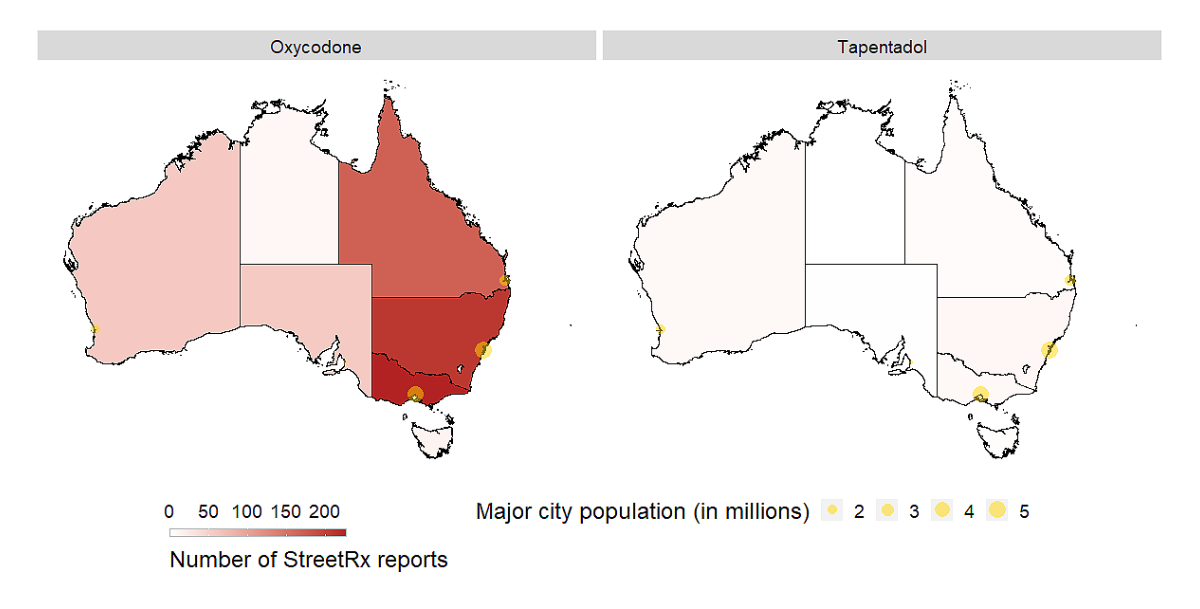
Geographic distribution of illicit sales in StreetRx. More illicit sales were reported for oxycodone than tapentadol, and reports were concentrated in states with larger city populations.

Most number of street sales was reported in New South Wales, Australia, and 6 states or territories had at least one sale reported (Figure 2). Nationally, there were 31 reports of tapentadol sales and 756 reports of oxycodone sales (Table 3), and reports were concentrated in states with larger cities. The geometric mean sale price per milligram for oxycodone (Aus $1.28 [US $0.91]/mg) was higher than that of tapentadol (Aus $0.12 [US $0.09]/mg). When stratified by release type, there was little difference in geometric mean price per milligram between extended-release and immediate-release tapentadol. The geometric mean price per milligram of immediate-release oxycodone was more than twice that of extended-release oxycodone. A total of 57.8% (436/756) of oxycodone sales were for immediate-release products; 16% (5/31) of tapentadol sales were for immediate-release products. The reasons for sale were generally similar between products. Notably, a higher proportion of sales of tapentadol was for self-treatment of pain or other medical conditions than for oxycodone. No report of a tapentadol sale included getting high as the reason for the sale; a total of 45 reports indicated this reason for oxycodone. However, a substantial portion of reports did not list a reason for the sale as the website users might have been more reluctant to enter sensitive or illegal use (such as to resell).

**Table 3:** Characteristics of street sales entered in the StreetRx website.

Variables				Tapentadol		Oxycodone
**Number of reports, n (%)**						
	**All reports**			31 (100)		756 (100)
		Extended-release	11 (35.48)		218 (28.84)	
		Immediate-release	5 (16.13)		437 (57.8)	
		Unknown	15 (48.39)		101 (13.36)	
**Geometric mean price per milligram, Aus $ (US$)**						
	**All reports**			0.12 (0.09)		1.28 (0.91)
		Extended-release	0.09 (0.06)		0.69 (0.49)	
		Immediate-release	0.10 (0.07)		1.84 (1.31)	
		Unknown	0.16 (0.11)		1.08 (0.77)	
**Reason for purchase, n (%)**						
	To prevent or treat withdrawal			0 (0)		10 (1.32)
	For enjoyment or to get high			0 (0)		45 (5.95)
	To resell			1 (3.23)		8 (1.06)
	To self-treat pain or another medical condition			14 (45.16)		219 (28.97)
	To come down			0 (0)		3 (0.4)
	Missing or did not report^a^			16 (51.61)		471 (62.3)

^a^Website users might skip this question, indicate they do not wish to answer, do not know the answer, or the question was not asked; the question was added in September 2016.

Regression models were used to test whether there were differences in street prices or the number of sales between tapentadol and oxycodone (Table 4). The first set of models were linear models that tested the differences in street prices. In a simplified model that used only the year of sale as a covariate, the effect between drugs was not significant (*P*=.67). However, in the simplified model, there were 9 influential points in the regression. Of these 9 points, 8 (89%) were from tapentadol, and many were at relatively high prices. These points strongly influence the estimate of the difference in price toward the null hypothesis (ie, no difference between drugs). Given the small number of observed points, removing these points would substantially hamper the power of the model. When adjustments for potency of the drug were added, the effect of the drug was still not significant (*P*=.34). Only the MME of drug sales significantly predicted the price. For a 10-MME increase in the strength of the drug, the illicit price increased by 7.8% (*P*<.001) after adjusting for the year and drug. The second set of models were Poisson models that tested the differences in the number of sales. The simplified model used only total website activity as a covariate, and the effect of the drug was significant (*P*<.001). However, when adjustments for licit availability were added, the effect of the drug was no longer significant (*P*=.98). The effect of standard units sold was significant (*P*=.03), but the effect on the number of illicit sale reports was relatively small. A 100,000 unit increase in standard units sold increased the likelihood of an illicit report by only 0.1%. Over the 3-year period, there were relatively few reports of sales, particularly for tapentadol. Notably, the second model to estimate the number of reports had only 10 tapentadol reports from 2016 to 2018. It is likely that both models are underpowered to detect meaningful differences between drugs in models with more covariates.

**Table 4:** Modeling differences in street price and number of sales.

Parameters				Model estimate		*P* value		Exponentiated parameter (95% CI)
**Differences in street price^a^**								
	**Simplified linear model**							
		Intercept	2.36		<.001		10.6 (6.93-16.3)	
		Drug (reference: tapentadol)	0.089		.67		1.09 (0.729-1.64)	
		Year	−0.032		.35		0.968 (0.904-1.04)	
	**Linear model with potency added**							
		Intercept	2.10		<.001		8.19 (5.36-12.5)	
		Drug (reference: tapentadol)	0.191		.34		1.21 (0.816-1.79)	
		Year	−0.0326		.33		0.968 (0.906-1.03)	
		10-MME^b^ increase	0.0754		<.001		1.08 (1.05-1.10)	
**Quarterly number of sales**								
	**Simplified Poisson model**							
		Intercept	−1.23		.004		0.294 (0.127-0.677)	
		Drug (reference: tapentadol)	3.99		<.001		54.2 (27.0-109)	
		Total website reports	0.00465		<.001		1.005 (1.003-1.007)	
	**Poisson model with availability added**							
		Intercept	−2.03		<.001		0.131 (0.0457-0.378)	
		Drug (reference: tapentadol)	0.0482		.98		1.05 (0.0289-38.1)	
		Total website reports	0.00557		<.001		1.006 (1.003-1.008)	
		100,000-unit increase in standard units sold	0.00882		.03		1.01 (1.00-1.02)	

^a^Street price, the dependent variable, was log-transformed for modeling.

^b^MME: morphine milligram equivalents.

## Discussion

### Principal Findings and Policy Implications

The results presented here indicate that Australians using the web perceive tapentadol as safer and less desirable for illicit activities than oxycodone. The overall sentiment of tapentadol posts tended toward promoting therapeutic use, implying that the population using the web uses tapentadol as intended more so than oxycodone. If reflective of the larger national population, the web-based content presented here could indicate that major consequences of addiction, overdose, death, and other adverse events are less common for those who use tapentadol than for those who use oxycodone. The conclusions were strengthened as multiple types of posts were analyzed. Collecting data from both forum-type sources and social media sources allows for more diverse discussion topics to be analyzed [9]. Both the low percentage of posts promoting abuse and the lack of reports of tapentadol street sales suggest there is little desire for tapentadol as a drug of abuse. As tapentadol is a drug with a mixed mechanism of action [15], there are pharmacological reasons that account for its lower desirability. We originally hypothesized that because tapentadol had a lower *μ-*opioid receptor affinity [16], there would be lower street prices once potency was accounted for. Neither the simplified model nor one with potency added detected significant differences between the drugs. Although no evidence was detected for the original hypothesis, once potency was added to the model, higher potency drug sales led to significantly higher prices. If higher prices indicate higher desirability, this suggests that a drug control policy that gives more attention to high potency doses could be effective in curbing market desirability. However, given the relatively recent approval of tapentadol, more familiarity among individuals who use drugs might increase the desirability (and therefore, the street price) independent of potency.

Control of prescription drug supply can improve health outcomes. In the United States, declining prescriptions for opioids has contributed to declining adverse health outcomes from prescription painkillers [17]. As diverted drugs are frequently found in overdose deaths [5], prevention of diverted supply could mitigate overdose mortality and other adverse outcomes. The difference in illicit market activity between the drugs suggests that differences in diversion control policies should be considered. Both substances are Schedule 8 drugs, but more nuanced policies with stricter controls on more desirable drugs could be more appropriate.

### Public Health Surveillance Implications

Ongoing pharmacoepidemiological surveillance of prescription opioid use and harms in Australia is scarce. A review found 15 reports from 2000 to 2018 [18]. The National Drug and Alcohol Research Centre produces annual reports on illicit drug harms [2], but information on prescription drugs is limited. Not all drugs have the same desirability, effect on the body, or potential for harm. Our study has reinforced the differences between the 2 opioids in illicit availability and web-based perceptions of safety, 2 factors that could influence eventual harm. Differences among other prescription drugs are likely to exist. Elucidating these differences on a broader scale than in this work would help identify the drugs best suited to have the highest benefit to those needing opioid pain relief and lowest risk to the Australian public. Given the scarcity of surveillance data, descriptive results derived from internet sources can be used as a primer for more complex approaches, such as system models quantifying trafficking or risky behaviors (eg, injection or concomitant use).

The approach to analyzing web content presented here used a systematic manual coding method combined with random sampling of the entire population of scraped posts, which presented several advantages. This avoided limitations that might arise when using natural language processing and machine learning to train on rare outcomes (such as drug tampering). Although internet posts can be ambiguous and lack context [19], manual coding is advantageous in identifying novel or rare content. Manual coding allowed for a large list of safety-related drug use topics, and this study is the first to characterize sentiment in terms of safe drug use, rather than as a form of approval [20]. Furthermore, the entire population of scraped posts was available for sampling, which eliminated several (but not all) sources of selection bias that might arise from smaller scale studies. Web posts were collected from all publicly scrapable Australian websites (including social media, blogs, and forums). Other approaches to internet surveillance tend to focus on social media, which could exclude certain types of content [9]. Sampling leverages frequentist methods for CIs, permitting valid inferences within the context of the sampling frame, even for rare outcomes. Finally, the overall approach presented here derived results from (1) unstructured web content and (2) structured, crowdsourced data entry. This combined study design allowed complementary interpretation of drug desirability using different methodological approaches.

### Limitations

The primary limitation of this study is that the sampling frames limit generalizability. The sampling frame of web posts does not include private websites (eg, private Reddit forums and Bluelight.org), which likely contain pertinent information. Australians also have access to forums outside the country, which could influence their opinions. If the keyword list was incomplete, then some posts would not be scraped, resulting in a selection bias. Illicit sales data are collected through crowdsourcing from the StreetRx website [21], and bias could exist between illicit sales reported to the website and the universe of all illicit sales. This would primarily cause the number of illicit sales to be underestimated because not all illicit sales would be entered into the website. Self-report data could also be subject to recall bias. Unless there is a differential bias in the underestimation between tapentadol and oxycodone, there would be a small impact on the comparative conclusions from this study. No information on the activities before the sale is available via the StreetRx website. Information describing causal elements that lead to diversion would be beneficial in crafting policies targeted toward drugs at the highest diversion risk. Finally, as an observational study, the number of reported street sales could not be controlled, leading to lower power to detect differences between drugs.

### Conclusions

Australians searching the web for opinions about drug use will generally view discussion of tapentadol as safer than oxycodone. Although strength and licit availability are significant factors in the illicit market, the illicit sales market for tapentadol was smaller than that of oxycodone.

## Appendix

Multimedia Appendix 1 Keywords for web content search.

## Abbreviations

MME: morphine milligram equivalents
