# Supplementary material for: Web-Based Discussion and Illicit Street Sales of Tapentadol and Oxycodone in Australia: Epidemiological Surveillance Study
Source: JMIR Public Health Surveill. 2021 Dec 20;7(12):e29187. doi: 10.2196/29187 (PMC8726044; doi:10.2196/29187)
Supplement: Multimedia Appendix 1 [file publichealth_v7i12e29187_app1.docx]

**Supplemental Table1. Search string criteria for keywords associated with tapentadol**

| **Keyword** | **Search String Criteria** |
| --- | --- |
| Nucynta | nucynta, nucinta, nucenta, nucyntas, nucytna |
| Palexia | palexia, palexea, palexias |
| Tapentadol | tapentadol, tapentadole, topentadol |

**Supplemental Table 2. Search string criteria for keywords associated with oxycodone**

| **Keyword** | **Search String Criteria** |
| --- | --- |
| Abtard | abtard |
| Alcet | alcet |
| Carexil | carexil |
| Combunox | combunox |
| Dazidox | dazidox |
| Endocet | endocet, endoset, endocets |
| Endocodone | endocodone |
| Endodan | endodan |
| Endone | endone, endones |
| ETH-Oxydose | eth-oxydose |
| Ixyldone | ixyldone, ixyldones |
| Leveraxo | leveraxo, leveraxos |
| Longtec | longtec, longtecs |
| Lynlor | lynlor |
| Lynox | lynox |
| Magnacet | magnacet |
| Myloxifin | myloxifin, myloxifins |
| Narvox | narvox |
| Novacodone | novacodone, novacodones |
| Onexila | onexila, onexilas |
| Oxaydo | oxecta, oxectas, oxaydo, oxaydos |
| Oxeltra | oxeltra |
| Oxyir | oxy.ir, oxy ir, oxy.irs, oxyir, oxyirs |
| Oxyargin | oxyargin, oxyargins |
| Oxycet | oxycet, oxycets |
| Oxycodone | oxycodone, oxicodone, oxycodon, oxykodone, oxycodones, oxycoden |
| Oxycodor | oxycodor, oxycodors |
| Oxycone | oxycone, oxycones |
| Oxycontin | oxycontin, oxycotton, oxycotin, oxyconten, oxycottin, oxycoton, oxycontins, oxy 40, oxy 80 |
| Oxyfast | oxyfast |
| Oxylan | oxylan |
| Oxylenus | oxylenus |
| Oxylieve | oxylieve, oxylieves |
| Oxyndone | oxyndone, oxyndones |
| OxyNeo | oxyneo, oxyneos |
| Oxynorm | oxynorm, oxinorm, oxynorms |
| Oxypro | oxypro |
| Percocet | percocet, percocets, percoset, perkaset, percaset, percaset, percets, percasets, perkocet, perkuset, perkucet |
| Percodan | percodan, perkodan, percoden, percodans |
| Percolone | percolone |
| Perloxx | perloxx |
| Primalev | primalev |
| Primlev | primlev |
| Proladone | proladone |
| Ratio-Oxycocet | ratio oxycocet, ratio oxycocets, ratio-oxycocet, ratio-oxycocets |
| Ratio-Oxycodan | ratio oxycodan, ratio oxycodans, ratio-oxycodan, ratio-oxycodans |
| Reltebon | reltebon |
| Remoxy | remoxy, remoxys |
| Renocontin | renocontin, renocontins |
| Rivacocet | rivacocet, rivacoset, rivacocit, rivacocets |
| Roxicet | roxicet, roxycet, roxiset, roxicete |
| Roxicodone | roxicodone, roxycodone, roxicodones |
| Roxiprin | roxiprin |
| RoxyBond | roxybond, roxybonds, roxibond |
| Shortec | shortec |
| Supeudol | supeudol, supuedol, supedol, supeudols |
| Targin | targin, targins |
| Targinact | targinact, targenact, targinict, targanact, targinacts |
| Targiniq | targiniq, tarqiniq |
| Troxyca | troxyca |
| Tylox | tylox |
| Xartemis | xartemis, xartemises |
| Xedone | xedone, xedones |
| Xolox | xolox |
| Xtampza | xtampza, xtampzas |
| Zomestine | zomestine |
